# Climate Change and Public Health in North Carolina: A Unique State Offers  a Unique Perspective

**DOI:** 10.1289/ehp.1408542

**Published:** 2014-06-01

**Authors:** Leah Devlin, Mikey Goralnik, William G. Ross, Kimberly Thigpen Tart

**Affiliations:** 1 University of North Carolina Gillings School of Global Public Health, Chapel Hill, North Carolina, USA; 2 City & Regional Planning Program, University of North Carolina at Chapel Hill, Chapel Hill, North Carolina, USA; 3 Landscape Architecture Program, North Carolina State University, Raleigh, North Carolina, USA; 4 Duke University Nicholas School of the Environment, Durham, North Carolina, USA; 5 Brooks, Pierce, McLendon, Humphrey & Leonard, LLP, Raleigh, North Carolina, USA; 6 Office of Policy, Planning, and Evaluation, National Institute of Environmental Health Sciences, National Institutes of Health, Department of Health and Human Services, Research Triangle Park, North Carolina, USA; E-mail: william.g.ross@duke.edu

Every year the Research Triangle Environmental Health Collaborative subjects to public and expert inquiry an issue that is important on a state, national, and global scale. For 2013, the issue chosen was public health and climate change. The “2013 Public Health and Climate Change Summit: Focusing North Carolina Forward” (http://environmentalhealthcollaborative.org/summit/summit-2013) convened 29–30 October 2013 at the North Carolina Museum of Natural Sciences (Raleigh, NC). The summit resulted in a set of recommendations that participants believe are key to helping North Carolinians and those living in other equally vulnerable places to address the risks of climate change—those that are already occurring and those that are likely to occur in the future.

At least two attributes make North Carolina well suited to host a summit on public health and climate change. First, the state has a distinct and varied geography that makes it a microcosm of climate change impacts in the United States. Second, the state is home to the Research Triangle region, which roughly includes Raleigh, Durham, and Chapel Hill (and the universities in each city) at the corners of the triangle and Research Triangle Park in the middle. The depth and breadth of environmental health expertise in academia, government, nongovernmental organizations and foundations, and private technological industries make the Research Triangle the epicenter of contemporary thinking on environmental health. The Collaborative was created to take advantage of this thinking and to focus it across disciplines on creative solutions to vital issues at the intersection of environment and health.

At the Climate Change Summit, stakeholders with wide-ranging and multidisciplinary expertise were invited to consider the connections between climate change and public health, and the implications for both North Carolina and regions beyond. Participants were asked to consider these connections generally and also in three contexts—rural, urban, and coastal—corresponding to specific categories of North Carolina geography/demography that we anticipate will experience climate change in unique ways. The goal of the summit was to foster a discussion with the richness and traction of place-based context but that might also yield broadly useful ideas for innovation and collaboration for the responsible management of public health risks from climate change.

A series of compelling plenary presentations from leading experts in climate change, public health, and science communications launched the summit. For example, key messages from the National Climate Assessment (http://www.globalchange.gov/ncadac) informed discussions: *a*) Climate change threatens human health, and those impacts are already underway in the United States; *b*) climate change will amplify existing nationwide health threats, especially for vulnerable communities; and *c*) preparedness and prevention can protect people from some of these threats, but given that national capacity to adapt to increasing threats may be limited in the future, early, preemptive action has the most impact potential, and responding to climate change provides opportunities to improve health and well-being in a variety of contexts, including energy, agriculture, and transportation. Key current climate change and health issues spotlighted for the Southeast, including North Carolina specifically, were health impacts from sea level rise, catastrophic events, decreased water availability, and an increase in frequency, intensity, and duration of extreme heat events.

One plenary presentation came from the social science perspective and illustrated the importance of effective communication in translating research into actionable policies. Research from the FrameWorks Institute (Lindland 2013) showed that redefining an issue using values such as ingenuity, innovation, and collaboration had the best effects on policy support. For example, taking into account cultural models and modifying and simplifying the narratives and causal chains that are used to describe climate change and its human health impacts can make climate science more likely to be taken seriously in people’s thinking, potentially generating more public buy-in for climate change as a public health issue.

A principal feature of this Climate Change Summit, and indeed of all of the Research Triangle Environmental Health Collaborative environmental health summits, was the convening of working groups, in this case, corresponding to the three identified geographic/demographic areas to separately consider the potential public health impacts of climate change on each area. The working groups were charged with reporting back to their participating colleagues what each group had determined are the most pressing climate change and health impacts within its specific context. In the same process, each working group also identified the most relevant gaps in knowledge and research for each impact. This knowledge was shared with the summit as a whole, which reviewed and assessed the findings and arrived at a consensus set of recommendations for actions that should be taken by various constituencies within North Carolina—government, academia, industry, and the public—both separately and in concert, to enable the state to respond appropriately to the threats to public health posed by climate change.

The complete set of recommendations has been published (Allen et al. 2014), but some of the most critical recommendations are as follows:

## Cross-cutting

The relationship between climate change and the frequency and severity of extreme weather events, such as coastal storms and increased riverine flooding, will be critical for the state, and land use planning and regulation will play a critical role in mitigating the public health impacts associated with such extreme weather events.
There is great variability of vulnerability to climate risks among North Carolinians, and climate change public health threats will not affect all individuals equally. Thus, environmental justice concerns are a significant gap in our current knowledge base of how to adapt to and prepare for climate impacts. The National Institute of Environmental Health Sciences, which is headquartered in the Research Triangle and works closely with area universities, is focusing much of its climate change research on increasing understanding of differences in vulnerability by prioritizing research exploring the impacts of heat and air pollution on elderly adults, pregnant women, children, and other vulnerable populations.
Effective and appropriate actions need to be identified and carried out to address risks related to heat stress, mental health vulnerability, and problems with water quality and availability.
To assess vulnerability to heat stress—a key health impact of climate change—across the state, the summit recommended integrating various large and relevant data sets, including the National Weather Service, employment data from the Bureau of Labor Statistics, and social vulnerability indices.

## Rural

Aspects of rural life that increase the risk of heat-related illness from a warming climate, such as outdoor work, lack of air conditioning, and isolation from cooling centers, are knowledge gaps in how climate change will affect rural areas compared with other areas; these gaps need to be filled.
More effective management of watersheds, rural wells, and aquifers for nutrient and bacterial contamination will require cooperation and innovative thinking on the part of farmers, agricultural extension workers, agribusiness, local water and sanitation officers, and others to protect rural water supplies and decrease the risk of increased gastrointestinal illnesses.

## Urban

Discussions of the public health impacts of weather-related disaster events, such as Hurricane Katrina, Superstorm Sandy, and Typhoon Haiyan, on urban areas led to a recommendation for a postdisaster focus on public health efforts that will reduce future risks from similar disaster events.
Throughout the region, but particularly in urban areas, it is critical to craft policy at all levels of government that connects climate change to air pollution and health, for example, by seeking the co-benefits of simultaneously mitigating climate change and air pollution.
The fast growth of populations in urban areas creates dense areas where more people and infrastructure may be exposed to the public health impacts of climate change. However, such scenarios also create opportunities for using the expertise of fields such as city and regional planning to minimize climate risks and mitigate public health impacts.

## Coastal

North Carolina’s extensive coast presents ripe conditions for heat stress in labor-intensive industries, the compounding effects of vulnerable coastal medical infrastructure, increased vector-borne diseases, and injuries and illness from increased coastal storms, all of which should be considered public health priorities.
Because of the role North Carolina’s coast plays in the state’s economy (e.g., fishing, real estate, tourism), heritage (e.g., communities, public parks, preserves), and security (military bases and ports), the state should support enhanced coordination between coastal planning and public health fields, and expand multidisciplinary research on problems, including sea level rise, that will likely impact coastal areas.

Chancellor Carol Folt of the University of North Carolina at Chapel Hill, who is an expert researcher in the impacts of climate change, added her voice to the summit by emphasizing the important role of the universities in fostering interdisciplinary thinking and a team-learning model when tackling complex scientific problems. She noted that North Carolina possesses a wealth of such institutional resources, but added that it will require the inclusion of not just those in academic disciplines but also those from other sectors, including government, business, and the public, to find creative solutions to the problems posed for public health by climate change, both in North Carolina and elsewhere around the world. We couldn’t have said it better ourselves.
